# Feedback-Mediated Upper Extremities Exercise: Increasing Patient Motivation in Poststroke Rehabilitation

**DOI:** 10.1155/2014/520374

**Published:** 2014-06-02

**Authors:** Maša D. Popović, Miloš D. Kostić, Sindi Z. Rodić, Ljubica M. Konstantinović

**Affiliations:** ^1^Laboratory for Experimental Psychology, Faculty of Philosophy, University of Belgrade, 11000 Belgrade, Serbia; ^2^Faculty of Electrical Engineering, University of Belgrade, 11000 Belgrade, Serbia; ^3^Clinic for Rehabilitation “Dr Miroslav Zotović”, 11000 Belgrade, Serbia; ^4^Faculty of Medicine, University of Belgrade, 11000 Belgrade, Serbia

## Abstract

*Purpose*. This proof-of-concept study investigated whether feedback-mediated exercise (FME) of the affected arm of hemiplegic patients increases patient motivation and promotes greater improvement of motor function, compared to no-feedback exercise (NFE). *Method*. We developed a feedback-mediated treatment that uses gaming scenarios and allows online and offline monitoring of both temporal and spatial characteristics of planar movements. Twenty poststroke hemiplegic inpatients, randomly assigned to the FME and NFE group, received therapy five days a week for three weeks. The outcome measures were evaluated from the following: (1) the modified drawing test (mDT), (2) received therapy time—RTT, and (3) intrinsic motivation inventory—IMI. *Results*. The FME group patients showed significantly higher improvement in the speed metric (*P* < 0.01), and smoothness metric (*P* < 0.01), as well as higher RTT (*P* < 0.01). Significantly higher patient motivation is observed in the FME group (interest/enjoyment subscale (*P* < 0.01) and perceived competence subscale (*P* < 0.01)). *Conclusion*. Prolonged endurance in training and greater improvement in certain areas of motor function, as well as very high patient motivation and strong positive impressions about the treatment, suggest the positive effects of feedback-mediated treatment and its high level of acceptance by patients.

## 1. Introduction

Paresis of upper extremities, which follows the cerebrovascular accident, is an impairment resulting with diminished quality of life of many hemiplegic survivors [1, 2]. Intensive exercise by the paretic arm has been demonstrated as a valuable treatment, resulting in improved functioning [3]. The exercise is most effective if it is task related; however, many patients in the acute phase cannot perform the task and they need to rebuild the movement capacity first. This rebuilding process is often assisted by electrical stimulation and rehabilitation robots, and, more recently, the training includes augmented feedback in virtual reality (VR) [4, 5]. The listed assistance provides patients with the opportunity for repetitive, intensive, and task-related practice [6].

The hypothesis behind the introduction of VR to rehabilitation comes from research findings that indicate patient motivation as highly important for therapeutic outcome [7]. Patient cooperation and satisfaction with a given treatment are essential in achieving successful rehabilitation results [8]. Motivation is, however, a multifaceted concept, which has been shown to be linked to several factors, including features inherent to the prescribed regimen, personality traits of the patient, physician, and therapist, and characteristics of the broader social environment [9]. Patients who take an active role in their rehabilitation process have been shown to be highly motivated, in contrast to those who believe the outcomes of therapy depend on fate, the institution, therapist, and the wider health system [10].

Several commercial games combining entertainment with exercise have been developed and are greatly accepted by the general population. Even though, in some cases, the use of such games has shown superior results in comparison to other types of recreational therapy [11], research has indicated that people with motor function problems may have difficulty playing commercial games out of the box [12]. This raises the prospect of games being designed specifically for rehabilitation. In most cases, the information regarding movements made in a 2D environment pertains to their temporal characteristics only [13]. However, the exercise related to motor reeducation needs to consider spatial characteristics of movement as well [14].

Being that gaming is renowned for its ability to provoke high levels of engagement and hold attention for long periods of time and that patient motivation is shown to be highly important in therapeutic outcome, the incorporation of virtual reality and interactive gaming into stroke rehabilitation treatments has been widely accepted in the past years. Today, there are several commercial devices for poststroke rehabilitation that employ video games, such as the Armeo (Hocoma AG, Zurich, Switzerland), the InMotion Arm (Interactive MotionTechnologies Inc, MA, USA), or the ReJoyce (Rehabtronics Inc, Edmonton, Canada). There is, however, limited evidence that the use of virtual reality and interactive video gaming may be beneficial in arm function improvement when compared with conventional therapy [11, 15, 16], and further studies are required to confirm these findings [17].

The aim of our study was to investigate the question of the effectiveness of video gaming in rehabilitation by addressing directly its effects on patient motivation, endurance in training, and improvement in motor function. For this reason, we developed a very simple gaming system with feedback and compared its effectiveness with the same exercise performed conventionally without feedback. We aimed to investigate the three above-mentioned components and their relationships, in order to further clarify the feasibility of video gaming in rehabilitation and possibly shed more light on the mechanisms involved.

We developed a feedback-mediated treatment, which uses gaming scenarios for relearning spatiotemporal movement characteristics and allows online and offline monitoring of both temporal and spatial characteristics of planar movements [18], as is the current research trend [19]. This treatment is a movement exercise, with visual feedback coming from the screen showing targets and pathways to be followed. The targets and pathways are interactively set to correspond to the abilities of the patient at any time during the treatment. The level of difficulty of the task is progressively increased, based on the success achieved in the game. The outcome measures are kinematic measures of trajectory smoothness, movement speed, and precision. Movement kinematics is increasingly being used to objectively describe and monitor changes in motor impairment and function in patients with neurological damage [20–22], as it may be used to describe the quality of the movement and the presence of compensatory motor patterns when compared to normative motor patterns [23].

The design of our feedback-mediated treatment is based on the principles of game design, which are shown in past research [24] to have particular importance to rehabilitation. The principles include meaningful play, setting an appropriate level of challenge, and handling failure adequately in rehabilitation gaming. Central to creating and maintaining meaningful play is the concept of feedback, the methods by which the game responds to the actions made by the player. Existing research findings suggest that visual and auditory feedback may enhance motor and functional performance [25]. In addition, concurrent feedback, knowledge of performance, knowledge of results, and explicit feedback may be central in achieving improved performance. The level of difficulty of a game is a major influence on how engaging the game is to play [24]. Burke et al. [26] suggest that failure in rehabilitation games should be handled conservatively. Being that engagement is a prerequisite for positive therapeutic outcome, all engagement should be rewarded at least initially.

We present here results from a randomized proof-of-concept study that included two groups of hemiplegic patients. We assessed the outcomes of a three-week exercise of affected arm manipulation. One group of patients exercised with video game feedback (feedback-mediated exercise—FME), while the other group (control) performed the same exercises without video game feedback (no-feedback exercise—NFE). The aim was to investigate the effectiveness of gaming on patient motivation and the achieved improvement of motor functions.

## 2. Method and Materials

### 2.1. Participants

The study was performed in the Clinic for Rehabilitation “Dr Miroslav Zotović” in Belgrade. Twenty poststroke hemiplegic patients were recruited from the inpatient population. They were selected based on the admission records, doctor's recommendation, and initial interview. Inclusion criteria were the following: poststroke hemiplegic confirmed by the imaging record (CT scan or MRI), age between 18 and 85 years, medically stable, capable of understanding instructions and communicating, and estimated 2D workspace (WS) of the affected arm between 20% and 50% of the full arm range. All patients signed the informed consent approved by the ethics committee of the Clinic for Rehabilitation “Dr Miroslav Zotović.” In this study, a computer program randomly assigned patients to the experimental (FME) and control (NFE) groups, with groups being equivalent in size.

### 2.2. Hardware

**Figure 1:**
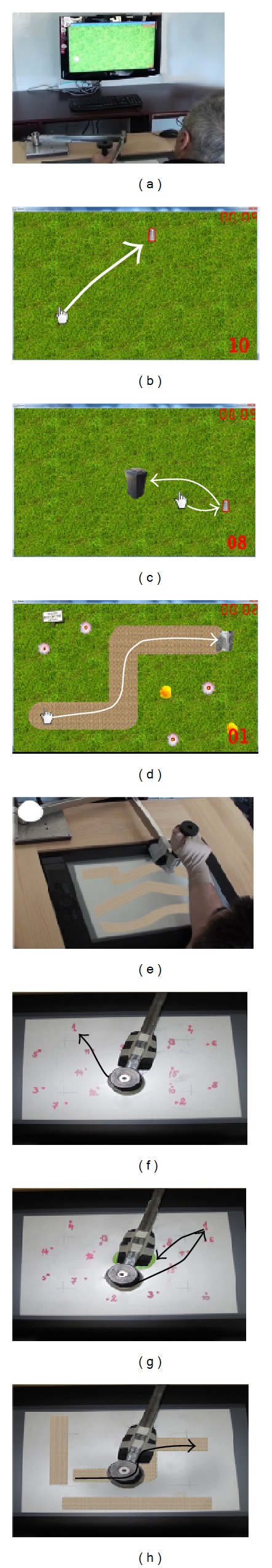
FME session (a) with screen shots of the first (b), second (c), and third (d) game, and NFE session (e) with photos of the first (f), second (g), and third (h) exercise. Examples of required movements are indicated with white arrows in each game or black arrows in each exercise.

The experimental hardware consisted of three major parts: the visualization system, the mechanical interface, and the signal recording system (Figure 1(a)). The center of the 22′ LCD screen was roughly in line with patient's eyes. The planar manipulandum was a custom-made mechanical rig with low inertia and virtually no friction. The rig consisted of two pieces and a handle attached to the open end of one rig's segment. The planar movement was performed by pushing/pooling the handle in various directions. The movement of the handle was recorded with the* Wacom Intuos XL* drawing board and cordless mouse attached to the rig's end. The workspace was limited to the board active area. The detailed description of the system is presented by Kostić et al. [18].

### 2.3. Procedure

Participants involved in this study received additional treatment five days a week, for three weeks. All patients received conventional therapy comprising one hour of physiotherapy, one hour of occupational therapy, and one hour of speech therapy (if needed), as is common for rehabilitation [27].

The additional treatment included exercise for upper extremities, which were different for the experimental and control group. Each such treatment session comprised three types of exercises that required the activity of the proximal joints (shoulder and elbow) of the affected arm. As suggested for additional exercise, the treatment lasted under thirty minutes [28]. Each of the three exercises lasted up to five minutes (actively), but patients were free to stop at any time if they felt fatigued. A five-minute rest period was set in between exercises, amounting to 25 minutes in total.

The minimum number of sessions was 13, and the three-week exercise period was extended if the patient missed more than two sessions for any reason.

Patients performed these tasks while holding the handle of the instrumented manipulandum, which recorded its position (Figure 1). They were seated in a chair with adjustable height, with their trunk secured by harness. Patients moved the manipulandum handle in order to perform the task.

*The FME* treatment used visual feedback via three-stage video game (Figure 1(a)). The input coordinates of the end-point (handle) controlled the game. The position of the cursor on the screen corresponded to the position of the handle on the board. During the game initialization, patient's 2D workspace was assessed, and the active area was scaled to ensure that the entire game window (full screen) represents the patient's WS. The score was displayed during the game to allow patients to follow their performance. A high-score list, where patients could compare their performance to previous days and to performance of other patients, was shown after each session. The therapist who was present in all sessions operated the game.

The first exercise required moving the cursor to reach targets, which were appearing in pseudorandom positions on the screen (Figure 1(b)). When the cursor would meet the target, it would disappear from the screen and trigger the “cash register” sound. This was followed by instantaneous appearance of the next target in a pseudorandom position on the screen. The minimal distance between two subsequent targets was defined as one-third of the WS diameter. The distance between targets gradually increased during the course of the game. The displayed score was the number of targets reached.

The second exercise required patients to move the cursor to a given target and then return to the initial position in order to trigger the next target appearance (Figure 1(c)). The initial position was in the center of the workspace, and the targets were appearing in the same manner as in the first exercise. In this exercise, as in the previous one, the score represented the number of reached targets.

In the third exercise, patients were presented with a target trajectory (path) which they had to follow with the cursor (Figure 1(d)). Upon completion of one path, a new, more difficult path would appear. The shape and complexity of paths (varying from wide and straight paths to narrow and complex paths) were designed by experienced therapists to match the abilities of patients. The score in this exercise represented the time of completion, but each transgression from the path was penalized with additional time.

*The NFE *group was instructed to perform the same exercises as the FME group but without the video game as a feedback. Here, target points or trajectories were presented on a paper sheet placed on the board surface under the manipulandum handle (Figure 1(e)). These sheets were replaced by the therapist who gave the instructions concerning which target sequence or path should be performed. For the NFE group there was no scoring by the computer, which eliminated the feedback of the success of the movements.

In the first two exercises (Figures 1(f) and 1(g)), patients were instructed to reach target points in the sequence numerated on the paper sheet. Upon completion, the therapist would replace the sheet with a more difficult one (with greater distance between targets). There were six different setups, with fifteen points, distributed following the same layout of the targets as in the FME group.

The trajectories in the third exercise (Figure 1(h)) were printouts of images used for the FME exercise. On each paper, there were three consecutive paths. Upon completion, the therapist would change the paper.

The same physiotherapists administered the FME and the NFE. The therapists who participated in the exercise sessions were instructed to operate the game or change task sheets, but not to provide additional feedback to patients.

A sample of these sessions has been video-recorded to allow later review if necessary.

Given the nature of the treatments, the study was single blinded (the outcome assessor was blinded to group allocation). Participants were requested to continue with their normal activities but not to participate in any other treatment related to upper extremities.

### 2.4. Outcome Measures

Outcome measures were assessed from (1) the modified drawing test—mDT, (2) received therapy time—RTT, and (3) intrinsic motivation inventory—IMI.

*mDT.* mDT was done at entry into therapy (baseline) and after intervention. In the mDT, participants were instructed to draw a square shape, based on the template appropriate for the patient's WS, as fast and as precise as possible, three times, and with a short pause in between [29, 30]. The best out of three performances, evaluated by minimizing criterion function for both speed and precision, was analyzed. Based on our previous work [18] and literature [31–33], we estimated patient progress by observing changes in movement speed, precision, and smoothness. Speed and precision were chosen as inherent to the test, while smoothness is a proven indicator of rehabilitation progress [31, 32]. These measures are presented in detail in [29]. 

*RTT*. RTT was defined as the time that patients spent exercising actively. This particular measure was used as it is suggested in literature to be highly correlated with motivation [34], and the purpose of our research was to investigate how the patient motivation to exercise is influenced by the presence of video games. A similar measure was used in other research on this subject [11]. All sessions during our study were timed, and RTT was followed continuously. The maximum score of this metric is 15 minutes. 

*IMI*. IMI is a multidimensional measurement device intended to assess participants' subjective experience related to a target activity in laboratory experiments [35, 36]. This questionnaire was used in our study to evaluate patient motivation for the received treatment. IMI has been used in several experiments related to intrinsic motivation and self-regulation, for example, in [37–41]. In recent years, the use of this questionnaire has spread in stroke rehabilitation studies [24, 42, 43]. The instrument assesses participants' interest/enjoyment, perceived competence, effort, value/usefulness, felt pressure and tension, and perceived choice while performing a given activity, thus yielding six subscale scores. The full scale consists of 45 items. For different research purposes, the IMI consists of varied numbers of items from these subscales, all of which have been shown to be factor analytically coherent and stable across a variety of tasks, conditions, and settings [44].

Following the instructions (http://www.selfdeterminationtheory.org/), we have constructed our own IMI questionnaire. From the full scale of 45 items, we have selected 26 items relevant to our study. The scale included all six subscales, with four subscales (perceived competence, effort, perceived choice, and felt pressure and tension) consisting of four items each and two subscales (interest/enjoyment and value/usefulness) consisting of five items (giving a total of 26 items). The original items in English have been translated into Serbian by a professional translator. Some items have been modified slightly to fit the specific activity being investigated. The items were randomized. The full list of items used in this study is given in Appendix A. 

*Interview*. After the questionnaires, a short structured interview, constructed for the purpose of this study, was conducted with each patient. Both groups of patients were asked to give their overall impression of the activity, as well as any suggestions concerning changes that could be implemented in order to make it more interesting for them. The interview with the FME group consisted of additional questions related to content of the rehabilitation treatment. The full list of questions included in the interview is presented in Appendix B.

### 2.5. Data Processing

*Movement Data Analysis*. Movements performed during mDT were analyzed with the following parameters.

1. *Speed* was calculated as the ratio of path length and time spent to complete the task.
2. *Precision* was estimated based on the quantity of the drawing outside of the square template. This procedure is described in detail by Kostić and Popović [29].
3. *Smoothness* was calculated based on characteristics of the velocity curve and jerk (third derivative of position), as described in [29].

Due to heterogeneity of both groups, no absolute measure could fairly evaluate effectiveness of the therapy. Therefore, measures are presented as coefficients of improvement. The improvement coefficient was calculated as the quotient of measure's postintervention score and the baseline score, in such manner that progress is reflected through increase of the measure (>1, improvement; = 1, no change; <1, regress).

*Patient Motivation Data Analysis.* The IMI questionnaire was given to all participants in both groups after three weeks of intervention. The patients filled out the questionnaires by stating their level of agreement/disagreement for each of the 26 items. Each item is evaluated on a 7-point scale (from 1 = not at all true to 7 = very true).

In order for the items on the IMI questionnaire to be scored, the first step is to reverse score the items marked with an R. These are items formulated as negative statements (see Appendix A; questions 4, 5, 9, 11, 13, 19, 23, and 24). This is done by subtracting the item score from 8 and using the resulting number as the item score. The next step is to calculate subscale scores by averaging across all of the items on that subscale. The subscale scores are then used in the analyses of relevant questions. Average subscale scores can range from 1 (lowest score) to 7 (highest score). 

*Statistics.* All variables were analyzed using the Statistical Package for the Social Sciences (SPSS) version 11.5 (SPSS Inc., Chicago, IL, USA). The Mann-Whitney test for independent groups was performed to test the significance of difference of movement metric scores, RTT, and IMI subscale scores between the FME and the NFE group.

Further, correlation was evaluated using Spearman's rank-order correlation coefficient, calculated between all IMI subscale scores (6) and mDT scores (3), as well as with RTT value, to examine the relationship between patient motivation and motor improvement.

Qualitative analysis was performed on data obtained in the short interviews conducted after completion of the IMI questionnaires.

## 3. Results

### 3.1. Participants

**Figure 2:**
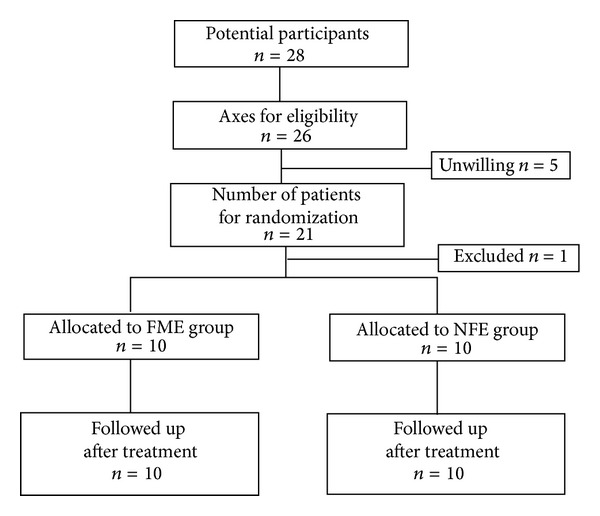
Consort diagram of study participant selection (feedback-mediated exercise—FME, no-feedback exercise—NFE).

Figure 2 presents the randomization process of participant selection (10 patients in each group).

During the participant selection process, one participant was randomly excluded from the 21 eligible participants who were willing to participate in the study, in order to achieve equal sample size in the two groups. The excluded participant was encouraged to engage in the exercise program but his performance scores were not recorded.

All patients completed the study. Patient demographics at study enrolment are presented in Table 1, including Fugl-Meyer (FM) score, upper extremities (UE) section [45]. Both groups were heterogenic in all aspects, therefore covering several segments of the population. However, there is no significant difference between the two groups in all variables.

### 3.2. Outcome Measures

Results of each test, for the FME and the NFE group, are presented in form of box and whiskers diagrams, depicting interquartile ranges. Red lines denote medians, while the bottoms and tops of the boxes denote the 25th and 75th percentile, respectively. 

**Figure 3:**
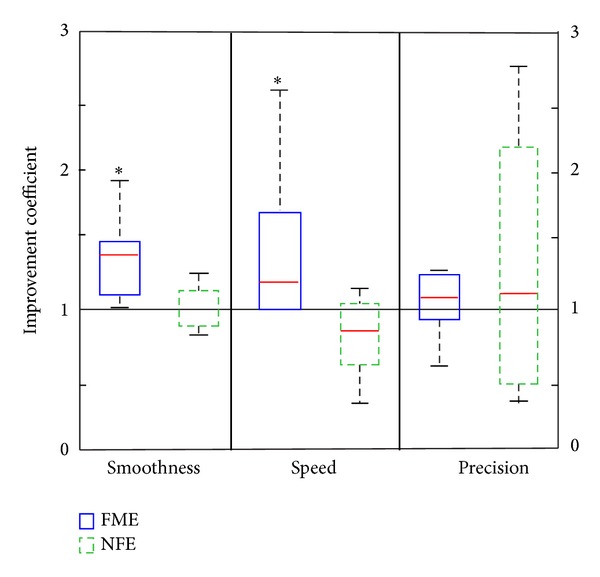
Box and whiskers plot of smoothness, speed, and precision improvement coefficients for FME and NFE groups. The black line denotes the improvement coefficient of 1 (equal result before and after treatment). Data with statistically significant difference is marked by *.

*mDT*. The improvement coefficients of smoothness, speed, and precision are shown in Figure 3. In the FME group, most participants show improvement (coefficient >1), except in precision where one quarter had improvement coefficients slightly lower than 1. The NFE group, on the other hand, shows less improvement or even regress in smoothness and speed, respectively, while in precision results are largely polarized, with one quarter of participants having improvement coefficients higher than 2 and one quarter lower than 0.5.

The smoothness improvement coefficients show a significant difference between the two groups with median 1.4 (IQR = 0.4) for FME and median 1 (IQR = 0.3) for NFE (*U* = 9, *P* < 0.01). The speed metric also shows significantly better progress in the FME group, with median 1.2 (IQR = 0.9), than in the NFE group, with median 0.8 (IQR = 0.5) (*U* = 10, *P* < 0.01). There is no difference in precision progress between the two groups (Md = 1.1, IQR = 0.6, for FME; Md = 1.1, IQR = 1.9, for NFE). 

**Figure 4:**
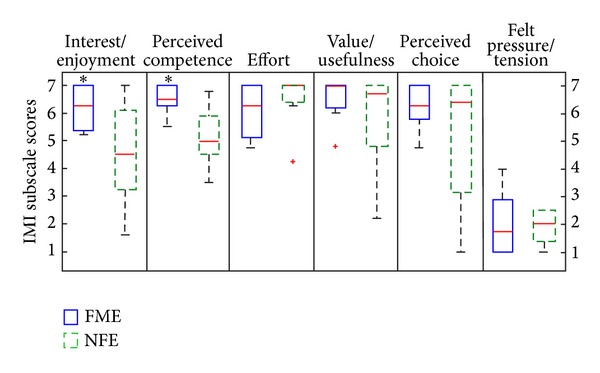
Box and whiskers plot of IMI subscale scores for FME and NFE groups; score range from 1 = not at all true to 7 = very true. Data with statistically significant difference is marked by *. Outliers are marked with a red +.

*IMI*. The results of the IMI questionnaire show high motivation in both groups related to specific rehabilitation treatment. The quartile distribution of subscale scores within each group is presented in Figure 4 in form of box and whiskers plot.

The results show significantly higher scores on the interest/enjoyment subscale in the FME group, with median 6.3 (IQR = 1.6), than in the NFE group, with median 4.6 (IQR = 3.3) (*U* = 14, *P* < 0.01). Significantly higher scores in the FME group are also observed on the perceived competence subscale with median 6.5 (IQR = 0.8) for FME and median 5 (IQR = 1.7) for NFE (*U* = 7.5. *P* < 0.01).

Very high average scores in the FME are obtained on the subscales effort (Md = 6.3, IQR = 2.1), value/usefulness (Md = 7, IQR = 0.9), and perceived choice (Md = 6.3, IQR = 1.5). The scores on these three subscales are shown not to be significantly different from the scores for the NFE group (Md = 7, IQR = 0.7; Md = 6.7, IQR = 2.7; Md = 6.4, IQR = 4.3, resp.).

The scores for the felt pressure/tension subscale are very low for both groups (Md = 1.8, IQR = 2.1, for FME; Md = 2, IQR = 1.3, for NFE) and no significant difference is shown between them. 

**Figure 5:**
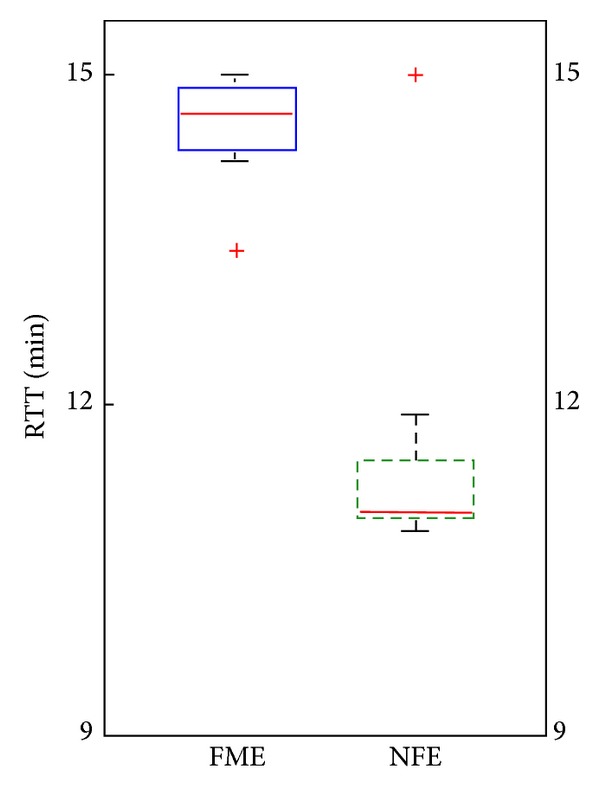
Box and whiskers plot of average RTT for FME and NFE groups; maximum RTT is 15 min. Outliers are marked with a red +.

*RTT*. RTT is the only measure followed continuously during the study. Both groups started out with relatively low therapy endurance (under 12 minutes, with no significant difference between the groups), but duration of exercising increased during the program. All patients in the FME group reached the metric maximum by the end of the first week and with few exceptions kept the maximal score throughout the program. The NFE group also improved but did not reach the metric maximum. Box and whiskers diagram of average RTT during the program for both groups is presented in Figure 5.

There is a significant difference between the two groups (*U* = 8, *P* < 0.01), with median RTT of 14.6 (IQR = 0.6) minutes for FME and median 11 (IQR = 0.7) minutes for the NFE group. 

*Correlation.* Spearman's rank-order correlation coefficients have been calculated to examine the relationship between metric scores and patient motivation. Results show significant positive correlation between scores on the perceived competence subscale and the smoothness metric scores (*r*_*s*_ = 0.69, *P* < 0.05), as well as positive correlation of perceived competence scores with patient speed improvement (*r*_*s*_ = 0.56, *P* < 0.05). The speed metric is also positively correlated with RTT (*r*_*s*_ = 0.63, *P* < 0.05). The other correlations are not statistically significant. 

*Qualitative Analysis*. Qualitative analysis of answers given by patients in the short follow-up interview has given valuable information about the FME rehabilitation treatment in its entirety. Sixty percent of patients in the FME group, when asked about adding specific content to the game, suggested that changing the existing task into a more interesting activity (e.g., playing a sport or arranging a garden) would be favorable. The same number of patients also favored adding music to the game (as was included in the third exercise during their treatment). Eighty percent of patients agreed that they would prefer having a reward/punishment effect added. Another aspect of the game discussed was competition during rehabilitation. Eighty percent of patients agreed that some form of competition is very important and increases their motivation in training. Type of competition preferred, be it with others or with themselves (or in some cases a preset standard), varied among patients. Analysis of patient comparison of the FME treatment to other conventional therapies included in their rehabilitation program shows that eighty percent of the patients find this form of treatment easier and more interesting. Overall, majority of patients in this group had a very strong positive impression of the intervention and they all stated they would continue using it at home if available. On the contrary, patients in the NFE group had mostly a vague impression of this additional exercise and considered it as a required segment of the entire rehabilitation program.

## 4. Discussion

The results of this study show a significant difference between FME and NFE in improvement of the speed metric, but no significant difference in the precision metric. This might be explained by the fact that the score achieved in the game by the patients in the FME group was directly dependent on the number of movements performed (reached targets or completed paths), motivating them to perform movements at the highest possible speed. This suggests that majority of FME group patients chose a competitive strategy, focusing on speed, rather than precision, in order to achieve higher scores. On the other hand, in the NFE group, the number of movements was not recorded, nor available for comparison with their own previous scores or results of others. As their movements were not timed, the prevailed strategy in performing movements was concentrating only on precision. Our results that show comparable improvement in precision in the FME and the NFE group, while having significantly higher improvement in speed in the FME, are in line with existing research findings suggesting that concurrent feedback, knowledge of performance, knowledge of results, and explicit feedback may be central in achieving improved performance [25].

A significant difference between the two groups was also shown in improvement of movement smoothness in favor of the FME group. This may be explained by the fact that smooth movements are more efficient as they require less energy and comprise less (if any) submovements [32, 33]. Such a result supports the assumption that the FME group adopted a competitive strategy. The tendency to achieve higher scores in the game is likely to result with development of motor strategies, which enable movements that are more efficient. Furthermore, strong correlation between smoothness improvement and perceived competence in our findings is in accordance with the premise that smoothness is one of the main characteristics of healthy arm movement [31–33].

The measure with the most evident difference between the groups, in favor of the FME group, is RTT (around 30%). This finding is important since there were no significant differences between the two groups of patients in FM score, age, nor time after stroke, prior to treatment. While exercising, especially during the initial phase of the program, patients of both groups complained of fatigue and discomfort and were convinced that they could not endure the full 15 minutes of exercise. Patients in the FME group were more willing to exercise in spite of these difficulties, and after several sessions, all of them reached the maximum exercise time. Further, there were no relapses, except on a few occasions, caused by unusual joint pains, which lasted one session. However, the NFE group rarely reached maximum and even then frequently relapsed. This difference between the two groups might be explained by the fact that only the FME patients were score-driven. This finding complies with the observed positive correlation of RTT and movement speed in our study. Our results are in line with the results reported in [11], where the total time of received intervention was around 6% higher for the group using motion-controlled video games, compared to the group receiving other forms of recreational therapy. The greater difference (around 30%) in our study may be explained by the fact that in [11] patients in the control group performed tasks which were less physically demanding than playing video games and were somewhat entertaining.

The Fugl-Mayer score was used in our study to assess patients on entry, in order to verify the balance between the two groups. Regardless of initial severity of motor deficit, the most dramatic recovery of motor function after stroke based on FM scores occurs within the first month [46]. Therefore, we did not expect any notable changes in FM score in our patients (FME group: 17 ± 14, NFE group: 20 ± 14 months after stroke) within 3 weeks of exercise, and consequently, the FM score was not used as an outcome measure.

Both groups of patients expressed a high level of motivation for this additional rehabilitation treatment. The FME group, however, shows significantly higher scores on the interest/enjoyment subscale compared to the NFE group. This subscale is considered the self-report measure of intrinsic motivation; thus, although the overall questionnaire is called the intrinsic motivation inventory, it is only the interest/enjoyment subscale that assesses intrinsic motivation per se [41]. Thus, we may assume that the gaming aspect of the rehabilitation treatment significantly increases patient motivation. Significantly higher perceived competence score of the FME group patients contributes to the positive aspect of the feedback-mediated treatment as well. The perceived competence concept is theorized to be a positive predictor of both self-report and behavioral measures of intrinsic motivation [41]. Patient awareness of their own ability to perform the task and awareness of performance improvement during the rehabilitation period are very important and overall contribute to patient interest in continuing treatment. This is supported by the positive correlation found between scores on perceived competence and both speed and smoothness improvement.

Our results on the IMI subscales of the FME group are in line with results obtained by Colombo et al. [42], who introduced the use of IMI to stroke rehabilitation research. Their study investigated whether robot-aided training boosts motivation and improves adherence. A 17-item scale version of IMI was applied at the end of a noncontrolled study, on 9 out of 12 patients treated with an elbow-shoulder rehabilitation device. In the correlation analysis between the parameters included in the evaluation metrics (patient's performance) and the motivation subscales of the IMI questionnaire, Colombo et al. [42] found a weak or no correlation in most relations and explained this as being in agreement with other reports in the literature. While they found that mean velocity was the only parameter showing a moderate correlation with both the effort and the pressure/tension subscales, we found speed significantly correlated with perceived competence and endurance in training.

Mihelj et al. [43] used a 25-item version of IMI in 16 stroke patients after exercise on HapticMaster in a VR scenario “The Message in a Bottle.” The results of the IMI showed a favorable response to this scenario on all subscales and the authors concluded that this VR scenario is highly motivating for patients and does not evoke pressure or tension.

Analysis of answers obtained through short interviews with patients in our study offers valuable suggestions on potential modifications and improvements of specific aspects of the feedback-mediated treatment. Findings from our interview suggest that a competition setting is extremely important for patients, with further elaboration on how much they have enjoyed competing during rehabilitation. The type of competition varied amongst patients; some preferred to compete with others, while others were more motivated by self-competition. This factor is already incorporated in the present gaming treatment by displaying the performance score of each patient on the computer screen.

Two more factors to be considered are difficulty levels of the game and reward/punishment effects. Both of these are shown in previous research [26] to be important principles of game design in rehabilitation. The level of difficulty of a game greatly influences the level of engagement of players. Among poststroke patients, the range of motor function is varied; thus, gaming rehabilitation treatments should offer a range of game challenges from which the patients can choose. The level of difficulty has been, to some extent, already included in our gaming treatment. However, some of the patients with better motor functions have expressed preference for even higher levels of challenge. The inclusion of the reward/punishment effects requires consideration of the possible negative effects of failure on patient engagement [24]. Although majority of patients in our study have stated preference of including these aspects to the treatment, punishment should be handled carefully, having in mind that all engagement in rehabilitation is important and better than having the patients withdraw from training. Based on our analysis, we may assume that patients' knowledge of performance scores, which potentially promotes a competitive strategy, is an efficient reward/punishment mechanism.

In summary, the results of this study show that, after three weeks of treatment, patients of the FME group displayed a higher motivation level and had longer endurance in training and better motor performance scores (movement smoothness and speed) compared to patients of the NFE group. Observing the relationship of these three measures, we found a correlation between the perceived competence aspect of patient motivation and improvement in movement speed and smoothness, as well as a positive correlation of the speed metric and endurance in training. Our findings may suggest that the underlying mechanism involved in the effectiveness of a feedback-mediated treatment may be the development of a competitive strategy, which motivates patients to exercise longer and achieve better motor performance.

Overall, a moderate significant effect in favor of virtual reality and gaming in poststroke rehabilitation in our findings is in line with results of other studies using upper limb retraining interventions [11, 15, 16].

The limitations of our study are the small size and heterogeneity of our patient sample, which may have influenced the statistical analysis and interpretation of the results. The fact that patients received the FME or NFE treatment as additional exercise combined with the conventional rehabilitation program may also have affected the results of this study.

Further comprehensive research in this area is needed, and our findings should be tested on larger patient samples. Randomized controlled trials should involve patients of different age groups, various levels of severity of stroke, and at numerous time points in their rehabilitation, in order to provide information about which patient groups would benefit the most from the proposed rehabilitation approach. Additional studies should investigate the benefits of feedback-mediated exercise received as full dose therapy, as well as the long-term effectiveness of such treatment.

Future development of virtual reality and gaming rehabilitation treatments should take into consideration the growing pool of knowledge acquired from patient evaluation of such treatments. Incorporation of patient suggestions into the game design may further increase their motivation for rehabilitation.

## 5. Conclusion

We developed a feedback-mediated treatment that uses gaming scenarios and allows online and offline monitoring of both temporal and spatial characteristics of planar movements.

In our study, we assessed the outcomes of a three-week exercise of the affected arm of poststroke hemiplegic patients. Overall, significant improvement in certain areas of motor function and prolonged endurance in training, as well as very high patient motivation and strong positive impressions about the treatment, suggest the benefits of feedback-mediated treatment and its high level of acceptance by patients, compared to no-feedback treatment.

## Figures and Tables

**Table 1:** Basic data of patients at study enrolment.

Age (Y)		Months after stroke		Fugl-Meyer for UE*	
FME	NFE	FME	NFE	FME	NFE
62	64	38	38	35	33
38	51	2	11	32	27
61	72	39	13	51	38
65	66	4	4	24	29
62	40	9	33	50	30
68	67	4	29	38	42
52	62	26	39	30	37
57	62	14	6	29	33
59	50	17	21	36	37
61	38	23	4	32	36

58 ± 8	57 ± 12	17 ± 14	20 ± 14	35 ± 8	34 ± 5

*FM for UE max score 66.

## Appendices

### A. Intrinsic Motivation Inventory (IMI)

The following statements express certain thoughts, beliefs, and feelings concerning the exercise you have been doing. Please indicate how true each statement is for you, using the following scale as a guide:

1  Not at all true
2
3
4  Somewhat true
5
6
7  Very true
(1) I enjoyed doing this activity very much.
(2) I think I am pretty good at this activity.
(3) I put a lot of effort into this.
(4) I did not feel nervous at all while doing this. (R)
(5) I thought this was a boring activity. (R)
(6) I believe this activity could be of some value to me.
(7) After exercising for a while, I felt pretty competent.
(8) I believe I had some choice about doing this activity.
(9) I did not try very hard to do well at this activity. (R)
(10) I think that doing these exercises is useful for recovery of my hand/arm movements.
(11) This activity did not hold my attention at all. (R)
(12) I felt very tense while exercising.
(13) I felt like it was not my own choice to do these exercises. (R)
(14) I thought this activity was quite enjoyable.
(15) I would be willing to do this again because it has some value to me.
(16) I am satisfied with my performance at this task.
(17) I think this is important to do because it can help me grasp desired objects more easily.
(18) It was important to me to do well at this task.
(19) I did this activity because I had no choice. (R)
(20) I was anxious while exercising.
(21) While I was exercising, I was thinking about how much I enjoyed it.
(22) I did this activity because I wanted to.
(23) This was an activity that I could not do very well. (R)
(24) I did not put much energy into this. (R)
(25) I felt pressured while doing these.
(26) I think doing these exercises could help me to recover faster.

The six subscales included the following questions:

Interest/enjoyment: 1, 5, 11, 14, 21
Perceived competence: 2, 7, 16, 23
Effort: 3, 9, 18, 24
Value/usefulness: 6, 10, 15, 17, 26
Perceived choice: 8, 13, 19, 22
Felt pressure and tension: 4, 12, 20, 25.

### B. Interview

1. Do you have any suggestions about changing this exercise in order to make it more useful and more interesting for you?
  Would you add something?
  Would you remove something?
2. What are your thoughts about including specific content to the game, for example, playing a certain sport or arranging a garden?
  Do you have any suggestions?
3. What did you think of the background music during one of the exercises?
  Did you enjoy it or did you find it disturbing?
4. What do you think about adding reward/punishment effects to the game (e.g., certain number of lives, more audio or visual effects)?
5. How do you feel about competing in this exercise?
6. Do you prefer self-competition or competition with others?
7. Would you continue to do this exercise on your own?
8. How did you experience this specific exercise in comparison to other rehabilitation treatments?
  Was it more or less difficult?
  Was it more or less interesting?
  Was it more or less useful?
9. What is your overall impression of this exercise?

The interview with the FME and the NFE group participants included the following questions:

FME: 1–8
NFE: 1, 6–8.
